# New Insights into Malignant B-Cell Disorders

**DOI:** 10.1155/2015/128084

**Published:** 2015-01-28

**Authors:** Marie-Christine Kyrtsonis, Kazuyuki Shimizu, Panayiotis Panayiotidis, Gerassimos A. Pangalis

**Affiliations:** ^1^Hematology Section of First Department of Propaedeutic Internal Medicine, University of Athens Medical School, Laikon General Hospital of Athens, Athens, Greece; ^2^The Japanese Society of Myeloma, Hematology Section of Internal Medicine, Tokai Chuo Hospital, Kakamigahara, Japan; ^3^Department of Haematology, Athens Medical Center, Psychikon Branch, Athens, Greece

Mature B-cell disorders comprise numerous and very different entities including all B-cell lymphomas (NHL) subgroups, chronic lymphocytic leukemia (CLL), and plasma cell dyscrasias [1].

Growing knowledge on disease biology has led to the development of new drugs, while innovative diagnostic techniques have resulted in improvement of diagnosis, establishment of new prognostic factors, and the recognition of new entities.

Original research and review articles published in this special issue highlight some interesting aspects of this topic. Herein, we will briefly present these papers and the scientific background they rely on.

The treatment of symptomatic multiple myeloma (MM), a severe plasma cell dyscrasia, with considerable morbidity and a reported, until recently, disappointing median survival of 3–5 years, has incredibly changed over the last decade, for the benefit of patients. Prolonged remission duration and improved survival with a better quality of life can be now observed, while the notion of an eventual cure does not seem so improbable anymore [2, 3]. The new drugs that led to the aforementioned improvements were not “chemotherapeutic” agents in the classical sense of the term because they were all “biology-modulating” factors. The first three “so-called” new drugs that constitute today the backbone of MM treatment are (i) bortezomib, the first-in-class reversible proteasome inhibitor that downregulates the three major disease expansion mechanisms, namely, cell escape from apoptosis, neoangiogenesis, and osteoclast activation and (ii) thalidomide and (iii) thalidomide-analog lenalidomide that both commonly exert antiangiogenic, anti-inflammatory, and immunomodulatory functions. Today, next generation novel agents are already available and others under evaluation, while treatment with monoclonal antibodies (mAbs) against disease specific targets has been developed and appears promising [4].

mAb treatment alone or in combination with chemotherapy (immunochemotherapy) begun much earlier for the treatment of B-NHL and CLL with the introduction in the late 90s of a humanized monoclonal antibody against CD20, rituximab, resulting in a significant improvement of patients' response rates. Afterward, intense research efforts were made and are still ongoing, to exploit the better knowledge of disease pathogenesis and to develop biologically active drugs in the field of B-NHL and CLL.

With regard to CLL, which is a relatively common leukemia of the elderly in western countries, it behaves indolently in more than half of patients but may have more aggressive behavior that should be immediately and effectively treated. Conventional therapy included alkylators, purine analogs, and corticosteroids, while the introduction of rituximab constituted a significant improvement. Numerous novel agents and mAbs are on their way, of which ibrutinib, an oral inhibitor of Bruton's tyrosine kinase, that promotes apoptosis and inhibits proliferation, migration, and adhesion of CLL cells [5] is already available. The same agent has shown activity in mantle cell lymphoma and other indolent B-NHL. Immunomodulatory agents, such as lenalidomide, that is currently routinely used for the treatment of relapsed MM showed also activity in relapsed/resistant CLL. In this context, it should be administered at much lower doses than in MM to avoid the dangerous flair phenomenon. The biologic background of new drugs activity is an interesting subject, and, intriguingly, variations in their mode of action may be observed among different B-cell entities.

Another therapeutic modality, high dose therapy with autologous hematopoietic stem cell (HSC) transplantation, although being a “classical and old” aggressive treatment option that can be applied to almost all B-cell disorders when it is for the benefit of patient to consolidate remission, had also improvements concerning stem cell mobilization. Knowledge of the regulation of HSC circulation and homing and adhesion to their surrounding milieu in the stem cell niche has led to the development of drugs that inhibit HSC adhesion and allow a better harvest.

Because of these therapeutic improvements, other fields of disease research had to move on. For example, the well-established prognostic factors and systems for MM, CLL, and B-NHL that mainly include respective staging, clinical and molecular adverse markers, could be shadowed in the era of biologic treatments. Reevaluation of these prognostic factors and the establishment of new ones is ongoing [6].

In the field of B-NHL, diffuse large B-cell lymphoma is the most frequently occurring entity and therefore has been better studied [7]. There are however some very rare entities, such as primary effusion lymphomas (PEL) and Waldenstrom's macroglobulinemia (WM), that remain largely understudied concerning the knowledge of their biology and optimal treatment's options. PEL-NHL is considered a very aggressive B-NHL caused by human herpesvirus type 8; it usually, but not always, occurs in HIV-infected patients. In HIV negative patients, its pathogeny is obscure, and in the absence of large series, there is no standard therapy [8]. Aggressive treatments have been administered to patients with conflicting results but also, in some instances, only drainage of the malignant effusions and pleurodesis with bleomycin (when the disease was located into the pleural cavity) produced prolonged remissions [9]. WM is an indolent lymphoplasmacytic lymphoma secreting an IgM component that may present resembling clinicolaboratory features and diagnostic overlap with other noncleaved small B-cell NHL without typical genetic features, such as marginal zone, mucosa associated, and small lymphocytic lymphomas [10]. Immunoglobulin heavy chain (IgH) gene rearrangement is the turning point of B-cell differentiation; likewise, IgH clonotypic sequence analysis may reveal the origin of the clone and/or preferential VH usage, helping differential diagnosis.

In addition, novel techniques and large multicenter studies have allowed the recognition of precursor B-cell disorders. It is now highly probable that all B-cell disorders are preceded by a premalignant condition. Few years ago, it became evident that all myelomas are preceded by monoclonal gammopathy of undetermined significance [11]. Then, “in situ” lymphomas were described. They are usually incidental findings and their risk of progression to clinically overt lymphoma is not known. Such lesions have been recognized for both follicular and mantle cell NHL and it could be assumed that they represent a preneoplastic condition that could regress by itself or on the contrary further evolve [12]. Another possible precursor condition of leukemic B-cell disorders, monoclonal B-cell lymphocytosis [13], is characterized by the presence of less than 5000 × 10^9^/L circulating clonal B-cells that can be CD5+ or CD5−. Usage of multiparameter flow cytometry has revealed that it is not a rare finding in the general healthy population [14]. Its significance remains to be defined.

In conclusion, we indeed realize that there is an enormous amount of new information concerning B-cell disorders which was not approached; we however hope that the aspects developed in this special issue will be appealing to the reader, either haematologist or physician or scientist involved in the field.

*Marie-Christine Kyrtsonis* *Kazuyuki Shimizu* *Panayiotis Panayiotidis* *Gerassimos A. Pangalis*

## References

[1] Swerdlow S. H., Campo E., Harris N. E. (2008). *WHO Classification Of Tumours Of Haematipoetic And Lymphoid Tissues*.

[2] Barlogie B., Mitchell A., van Rhee F., Epstein J., Morgan G. J., Crowley J. (2014). Curing myeloma at last: defining criteria and providing the evidence. *Blood*.

[3] Ludwig H., Sonneveld P., Davies F. (2014). European perspective on multiple myeloma treatment strategies in 2014. *Oncologist*.

[4] Ocio E. M., Richardson P. G., Rajkumar S. V. (2014). New drugs and novel mechanisms of action in multiple myeloma in 2013: a report from the International Myeloma Working Group (IMWG). *Leukemia*.

[5] Byrd J. C., Furman R. R., Coutre S. E. (2013). Targeting BTK with ibrutinib in relapsed chronic lymphocytic leukemia. *The New England Journal of Medicine*.

[6] Kanakry J. A., Ambinder R. F. (2014). Old variables, new value: a refined IPI for DLBCL. *Blood*.

[7] Martelli M., Ferreri A. J., Agostinelli C., Di Rocco A., Pfreundschuh M., Pileri S. A. (2013). Diffuse large B-cell lymphoma. *Critical Reviews in Oncology/Hematology*.

[8] Klepfish A., Zuckermann B., Schattner A. Primary Effusion Lymphoma (PEL) in the absence of HIV infection—clinical presentation and management.

[9] Yiakoumis X., Pangalis G. A., Kyrtsonis M. C. (2010). Primary effusion lymphoma in two HIV-negative patients successfully treated with pleurodesis as first-line therapy. *Anticancer Research*.

[10] Pangalis G. A., Kyrtsonis M. C., Kontopidou F. N. (2005). Differential diagnosis of Waldenstrom's macroglobulinemia and other B-cell disorders. *Clinical Lymphoma*.

[11] Landgren O., Kyle R. A., Pfeiffer R. M. (2009). Monoclonal gammopathy of undetermined significance (MGUS) consistently precedes multiple myeloma: a prospective study. *Blood*.

[12] Carbone A., Santoro A. (2011). How I treat: diagnosing and managing “in situ” lymphoma. *Blood*.

[13] Parikh S. A., Kay N. E., Shanafelt T. D. (2013). Monoclonal B-cell lymphocytosis: update on diagnosis, clinical outcome, and counseling. *Clinical Advances in Hematology and Oncology*.

[14] Shim Y. K., Rachel J. M., Ghia P. (2014). Monoclonal B-cell lymphocytosis in healthy blood donors: an unexpectedly common finding. *Blood*.

